# Intermediate magnetization state and competing orders in Dy_2_Ti_2_O_7_ and Ho_2_Ti_2_O_7_

**DOI:** 10.1038/ncomms12592

**Published:** 2016-08-25

**Authors:** R. A. Borzi, F. A. Gómez Albarracín, H. D. Rosales, G. L. Rossini, A. Steppke, D. Prabhakaran, A. P. Mackenzie, D. C. Cabra, S. A. Grigera

**Affiliations:** 1 Instituto de Física de Líquidos y Sistemas Biológicos (IFLYSIB), UNLP-CONICET, La Plata 1900, Argentina; 2 Departamento de Física, Facultad de Ciencias Exactas,Universidad Nacional de La Plata, 1900 La Plata, Argentina; 3 Instituto de Física de La Plata, UNLP-CONICET, 1900 La Plata, Argentina; 4 School of Physics and Astronomy, University of St. Andrews, St. Andrews KY16 9SS, UK; 5 Max Planck Institute for Chemical Physics of Solids, Nöthnitzer Str. 40, Dresden, Germany; 6 Department of Physics, Clarendon Laboratory, University of Oxford, Parks Road, Oxford OX1 3PU, UK

## Abstract

Among the frustrated magnetic materials, spin-ice stands out as a particularly interesting system. Residual entropy, freezing and glassiness, Kasteleyn transitions and fractionalization of excitations in three dimensions all stem from a simple classical Hamiltonian. But is the usual spin-ice Hamiltonian a correct description of the experimental systems? Here we address this issue by measuring magnetic susceptibility in the two most studied spin-ice compounds, Dy_2_Ti_2_O_7_ and Ho_2_Ti_2_O_7_, using a vector magnet. Using these results, and guided by a theoretical analysis of possible distortions to the pyrochlore lattice, we construct an effective Hamiltonian and explore it using Monte Carlo simulations. We show how this Hamiltonian reproduces the experimental results, including the formation of a phase of intermediate polarization, and gives important information about the possible ground state of real spin-ice systems. Our work suggests an unusual situation in which distortions might contribute to the preservation rather than relief of the effects of frustration.

## Abstract (web summary)

A classical Hamiltonian captures key properties of spin ice materials such as residual entropy and fractionalized excitations. Here, the authors present experimental results of the polarization transition that motivate a Hamiltonian with lattice distortions, which predicts an intermediate magnetization state and competing ground state orders.

## Graphical abstract

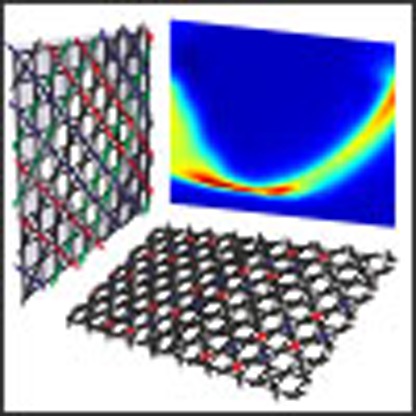

Spin-ice systems owe their name to an analogy of their ground-state construction rules to those in Pauling's model for proton disorder in water ice^1^,^2^. In their simplest variant, they can be described in terms of centre pointing classical Ising spins at the binding points of a network of corner-shared tetrahedra forming a pyrochlore lattice (see Fig. 1a). In real materials, such as Dy_2_Ti_2_O_7_ (DTO) and Ho_2_Ti_2_O_7_ (HTO), this is an accurate description for the low temperature behaviour. It was recognized early that, given the large magnetic moments of the rare-earth ions (of the order of 10 μ_B_) in addition to a ferromagnetic nearest neighbour coupling *J* (of the order of 1 K), a dipolar term was necessary in the Hamiltonian to describe the experimental results^3^,^4^,^5^. The minimal model that became the norm in the description of spin-ice materials is the standard dipolar spin-ice model (s-DSM):


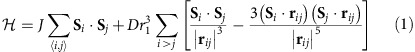


where *r*_1_ is the nearest-neighbour distance, **r**_*ij*_ is the distance between spins *i* and *j*, **S**_*i*_ is a classical spin of unit length, and the dipolar constant, *D,* is of the same order than *J*. Spin-ice systems belong to the class of geometrically frustrated magnets. Frustration is possible in other ways, but the class of geometrically frustrated materials has the advantage of better experimental control and theoretical description than the case where disorder is needed. An inherent weakness of geometrical frustration is that it might be relieved by distortions to the lattice^6^,^7^,^8^. The spin-ice materials DTO and HTO are fairly robust to distortions; experimentally, the properties of the frustrated state and its low-lying excitations are seen to dominate the intermediate temperature regime^9^. However, as we shall see, the effects of small distortions in this system might account for some specific experimental features and determine the presence or absence of an ordered state at low temperatures.

The different limits of the classical s-DSM already contain the most singular and attractive features of spin-ice materials, notably residual entropy, emergent gauge structure, fractionalized monopolar excitations^10^,^11^,^12^, and two-dimensional and three-dimensional Kasteleyn transitions^13^,^14^,^15^. The s-DSM also predicts an ordered state at very low temperatures^16^. As the field developed, it was soon recognized that additional interaction terms are needed to properly account for the experiments and an effort was made to determine an empirical Hamiltonian from the experimental data^17^,^18^. In particular, the most complete work of this type is that of Yavors'kii *et al*.^19^. In their approach, Monte Carlo simulations of a model with dipolar interactions, and isotropic first (*J*_1_), second (*J*_2_) and third (*J*_3_) nearest-neighbour exchange interactions are compared with DTO experimental susceptibility, specific heat and magnetization data for zero magnetic field *H*^20^,^21^,^22^, with *H* applied in the 112 direction^23^,^24^, and with the empirical dependence of its polarization transition with *H*//111^25^. By means of this comparison they put bounds to the values of *J*_1_, *J*_2_ and *J*_3_ and arrive at an empirical Hamiltonian, the generalized dipolar spin-ice model (g-DSM). The g-DSM gives a very good phenomenological description of several of the spin-ice features and reproduces the low temperature neutron scattering pattern of DTO noticeably better than the s-DSM^19^.

Despite its successes, the g-DSM model does not fully reproduce the low temperature behaviour of the spin-ice materials. In this work we address two instances of these, which were unknown at the time the model was developed: the doubling of the polarization transition with fields in the neighbourhood of 111 (refs 26, 27) and the recent report of an upturn in the specific heat below 0.6 K^28^. Based on our experimental results and those in the literature, we construct a new spin-ice model and show that in addition to reproducing the experimental features, it predicts an intermediate magnetization state for fields near [111] and the existence of different types of possible order at low temperatures when no field is applied.

## Results

### Magnetic susceptibility

We start by discussing the magnetic susceptibility at low temperatures when an external constant field is applied in the neighbourhood of the crystallographic [111] direction. For our experiments, single crystalline samples of DTO and HTO were used. The a.c. susceptibility was measured in a dilution refrigerator with an external field applied using a triple-axis vector (see Methods section).

**Figure 1:**
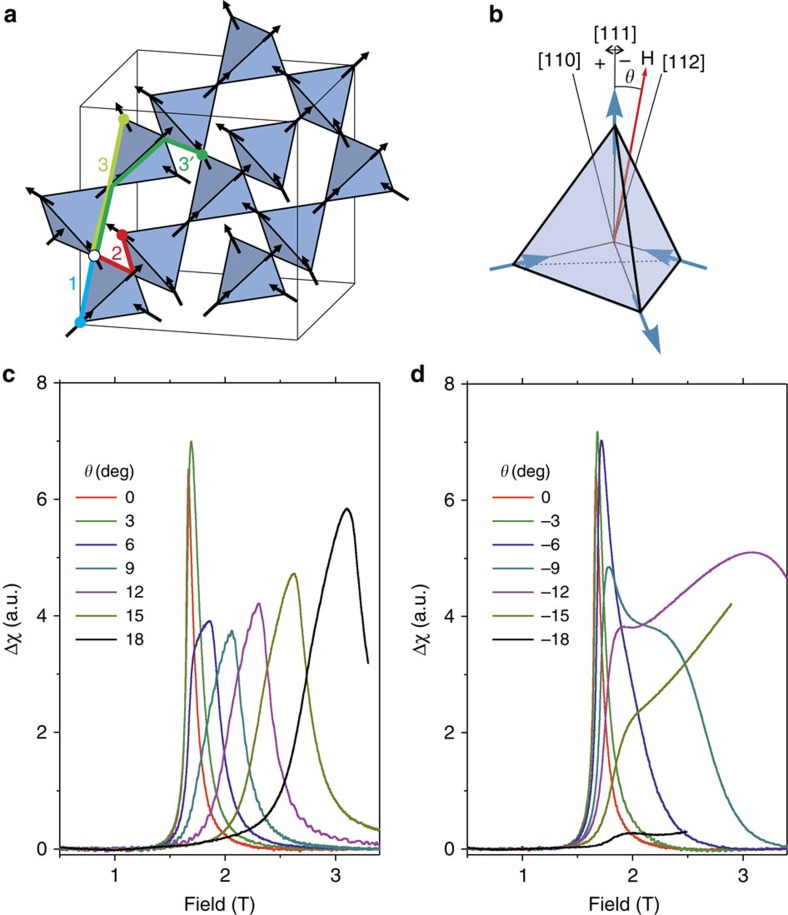
Magnetic susceptibility at the polarization transition. The data shown are from HTO, but are representative of both materials. (**a**) A schematic view of the pyrochlore lattice: spins are represented as black arrows, and tetrahedra are coloured light blue. The coloured lines show, starting from the white circle, a first (light blue), second (red) and two types of third (green and light green) nearest neighbour. (**b**) Sign convention for the angle shown in a single tetrahedron: rotations towards [112] are negative while those towards [110] are positive. (**c**) Magnetic susceptibility: when the field is rotated towards [110] the peak remains unique. (**d**) As the field is rotated towards [112], the peak splits into two, the upper one moves faster with field as the angle is increased. The temperature for all curves is fixed at 0.18 K and in all cases a smoothly varying background has been subtracted for clarity.

**Figure 2:**
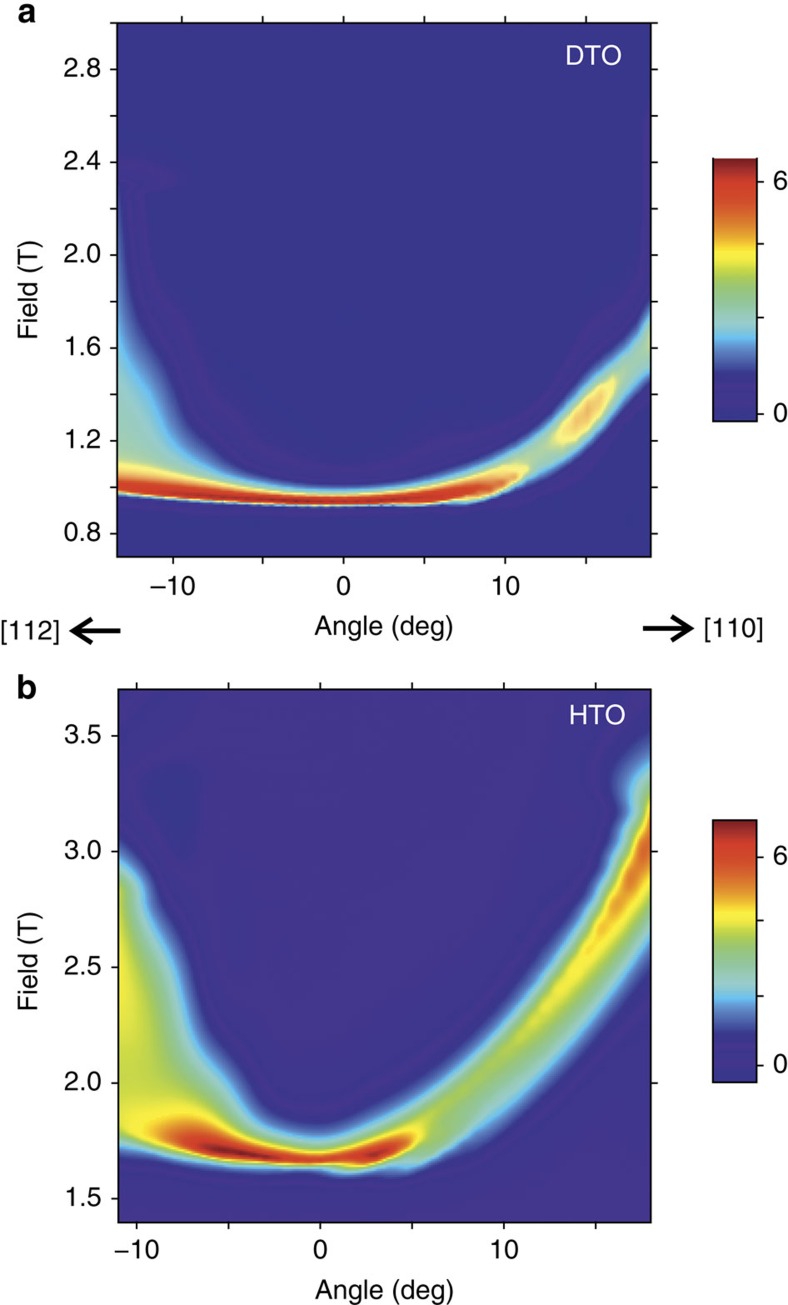
Contour plots of the magnetic susceptibility for DTO and HTO. (**a,b**) Interpolated contour plot of the magnetic susceptibility, for DTO and HTO respectively, at *T*=0.18 K. Each graph is constructed based on approximately 40 traces of *χ* versus angle at fixed temperature and field. Positive (negative) angles correspond to a rotation towards [110] ([112]). For both materials the transition widens—eventually resolving into two peaks—as the field is rotated towards [112]. The effect is more pronounced for HTO. The origin of this difference could lie on the non-Kramers nature of Ho^3+^ which makes it more susceptible to its local environment.

The change in a.c. susceptibility at the polarization transition is plotted in Fig. 1 for the HTO single crystal (analogous behaviour is seen in the DTO sample) as a function of field at a fixed temperature of *T*=0.18 K for different angles from the [111] direction (see Fig. 1b). Figure 1c shows field rotations from [111] towards [110], while Fig. 1d shows rotations towards [112]. The usual spin-ice models predict a single transition between the partially frustrated kagome-ice state at low fields and the polarized state at high fields. In the experiments, this is true when the field is perfectly aligned with [111], or when it is rotated towards [110], but the transition splits into two peaks, with an intermediate polarization region, when the field is rotated towards [112]. This behaviour is seen more clearly in Fig. 2, where the change in susceptibility is shown for both materials as an interpolated contour plot. In this figure, it is also quite noticeable that the angular dependence of the lower transition field is quite small when the field is rotated towards [112]. Similar behaviour had been previously observed in magnetization experiments in DTO^26^.

The failure of the usual spin-ice Hamiltonians to reproduce an experimental feature common to both spin-ice materials suggests that there is an additional physical mechanism that is being neglected in the theoretical treatment. In the following, we argue that a missing ingredient can be the effect of distortions in the magnetic couplings of the pyrochlore lattice.

### The role of distortions

We analyse how the s-DSM Hamiltonian (equation (1)) is modified in the presence of distortions. We consider the simplest case: classical Einstein phonon modes in the pyrochlore lattice, parameterized as **u**_*i*_*≈***r**_*i*_*−***r**_*i*_^0^, with a linear dependence of the exchange couplings on distances: *J*(**r**_*ij*_)*≈J*_*0*_[1−*α*·**r**_*ij*_^*0*^*·*(**u**_*j*_*−***u**_*i*_)], where *J*_0_ is the undistorted exchange constant and *α* is the spin-phonon coupling constant, and a simple quadratic term for the energy cost of distortions. Expanding the variation of 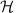 up to linear order in **u**_*i*_, one obtains a magnetoelastic Hamiltonian that can be written compactly as:


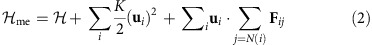


where *K* is the elastic constant for classical Einstein phonons, *N*(*i*) stands for the neighbours of site *i* and **F**_*ij*_ encodes the quadratic spin interactions between a site *i* and a neighbour *j*.

An effective Hamiltonian can be obtained from 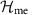 by integrating out the phononic d.f.^29^ (see Methods section). This effective Hamiltonian can be cast into a simple form where the effect of distortions to the lattice is translated into a change in the nearest-neighbour interaction and the emergence of further neighbour interactions. These interactions have their origin in the local changes in the magnetic interaction due to changes in the relative positions of the lattice sites, and are thus strongly dependent on the lattice connectivity between neighbours. Further neighbours of equal distance but connected to the original site by a different number of lattice sites develop different interaction terms. In the case of the pyrochlore lattice (see Fig. 1a), this mechanism provides two kinds of third nearest neighbour interactions, *J*_3_ and *J*_3_′.

### The model

While it cannot be expected that this simple analysis based on classical phonons will give accurate values for the effective exchange constants of the real system, which consists of a far more complicated lattice once all the non-magnetic atoms are taken into account, it provides the key observation that, regardless the specific material, further neighbour interactions should be considered and distinction made between differently connected neighbours of the same order, even if these terms or differences were negligible from the exchange integrals. With this information, we constructed a model to third nearest neighbours that incorporates these distinctions, the distorted dipolar spin-ice model (d-DSM), and explored it using Monte Carlo simulations (see Methods section and Supplementary Note 1). As additional constraints, we keep *J*_2_ and the mean value (*J*_3_*+J*_3_′)/2, within the first set of intervals determined by Yavorsk'ii *et al*. for the g-DSM^19^ to retain compatibility with the experimental results this model was fitted to (see also Supplementary Note 2 and Supplementary Fig. 1).

**Figure 3:**
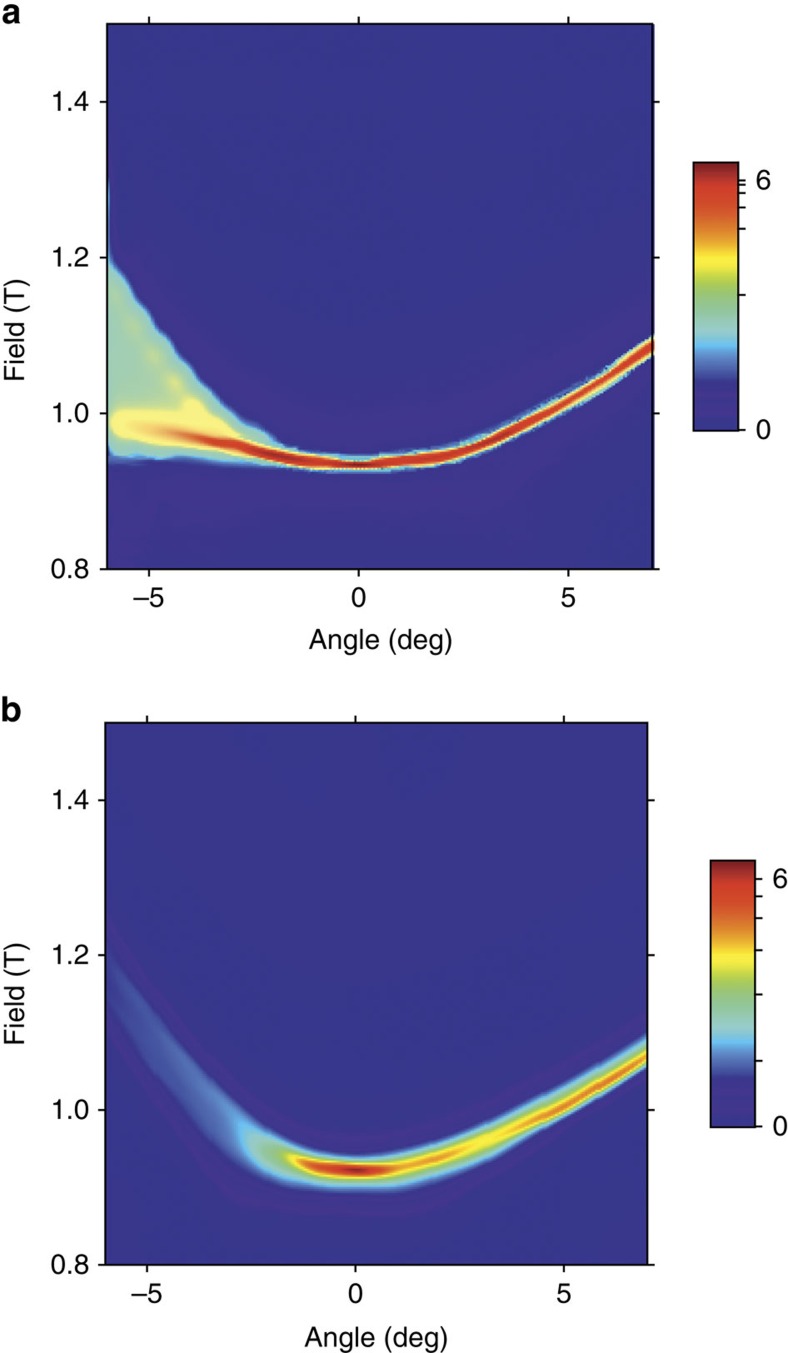
Magnetic susceptibility from Monte Carlo simulations. (**a**) Contour plot of the susceptibility as extracted from the d-DSM, which shows a doubling of the transition and a marked anisotropy similar to that seen in the experiments (compare with previous data and ref. 26). (**b**) The best possible fit to the data achievable with the g-DSM. In both cases the colour scale is non-linear to avoid saturation from the high intensity peaks.

Figure 3 shows the magnetic susceptibility for the d-DSM, with *J*_1_=3.41 K, *J*_2_=0, *J*_3_*=−*0.02 K, *J*_3_*′=*0.07 K and *D=*1.3224 K (Fig. 3a), and for the g-DSM model (Fig. 3b), both as interpolated contour plots. Unlike the s-DSM or g-DSM, and in keeping with the experiments, the susceptibility calculated in the d-DSM has a flatter angular dependence of the critical field for rotations towards [112], showing a broad peak that eventually resolves into two peaks, and remains unique when the field is rotated towards [110], all of which is in correspondence with the behaviour shown in Fig. 2 for the experimental results in the neighbourhood of [111]. Figure 3b shows the best possible fit to the data using the g-DSM: by forcing the parameters towards the higher end of the intervals given in ref. 19, it is possible to eventually achieve a small doubling of the transition when rotating towards [112], but it completely fails to reproduce the angular dependence of the critical field. The addition in the d-DSM of different types of *J*_3_ is therefore necessary to be able to reproduce the susceptibility experiments close to [111], even at a qualitative level. Further terms and analysis are required to reproduce in detail the full angular dependence.

### Intermediate phase

**Figure 4:**
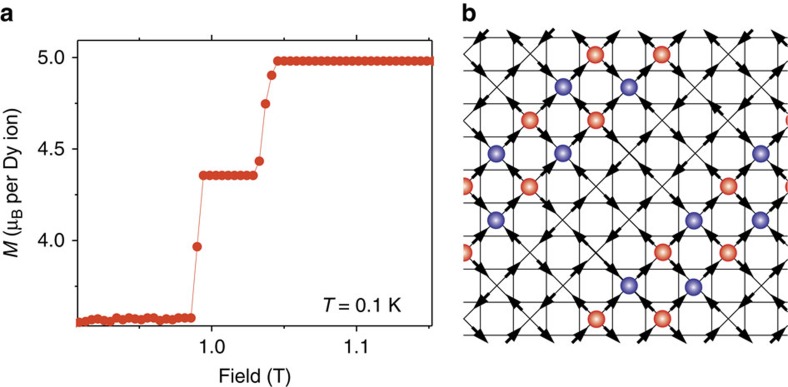
Plateu in the magnetization and intermediate state of the model with distortions (d-DSM). (**a**) The simulated magnetization curve in a homogeneous system as a function of field for *T*=0.1 K with the field at an angle of −5° from [111]. A mid-plateau is stabilised between the kagome-ice and the fully polarized state. (**b**) A sketch of this intermediate state projected along [100]. The coloured circles mark tetrahedra where the ice-rule is broken either by excess (blue) or defect (red) of inward pointing spins.

A known consequence of the coupling between spin d.f. and distortions is the stabilization of plateaux in the magnetization (see for example, refs 30, 31). It therefore comes as little surprise that the doubling of the polarization transition corresponds, in the limit of low temperature and perfectly homogeneous tensions, to the presence of a plateau between the kagome-ice and the fully polarized state (see Fig. 4a). This plateau marks an intermediate ordered state where the ice rule is broken in only half of the tetrahedra. To visualize these states, it is helpful to mark by a red or blue dot the defect or excess of inward pointing spins. Defects of different colour interact approximately as charges of opposite sign^11^. Full polarization corresponds in this picture to a complete checkerboard pattern of blue and red dots. The intermediate states of Fig. 4 are easily pictured as alternate arrangements of stripes of the checkerboard pattern separated by empty stripes (see Fig. 4).

### The ground state

One of the remarkable predictions of all dipolar spin models is that, contrary to initial expectations, spin-ice materials may have no extensive residual entropy even in the absence of an applied magnetic field. As shown by Melko *et al*.^16^, the introduction of dipolar interactions leads to a sharp transition at ∼0.18 K into an ordered ground state. At present there is no direct experimental determination of the ground state of spin-ice systems, since below 0.6 K the characteristic relaxation time of the system seems to grow faster than exponentially^32^,^33^ and most measurements probe properties of the system out of equilibrium^34^. A recent experiment^28^, in which the zero field low temperature specific heat was measured over much longer timescales, reports the possible observation of a peak in the specific heat that might correspond to the onset of order in DTO. However, the temperature at which this onset is seen (∼0.6 K) is not compatible with the prediction of Melko *et al*. for the s-DSM. The situation is very similar for the g-DSM: the additional interaction terms of this model leave the ordered state unaltered, and the specific heat (by construction), shows no upturn down to 0.2 K.

The model presented in this work (the d-DSM) is a different scenario altogether. In the simple homogeneous case, and within the constraints on the parameters imposed by the experiments (crucially our susceptibility near [111]) two different ordered ground states are possible depending on the relation between the exchange constants.

**Figure 5:**
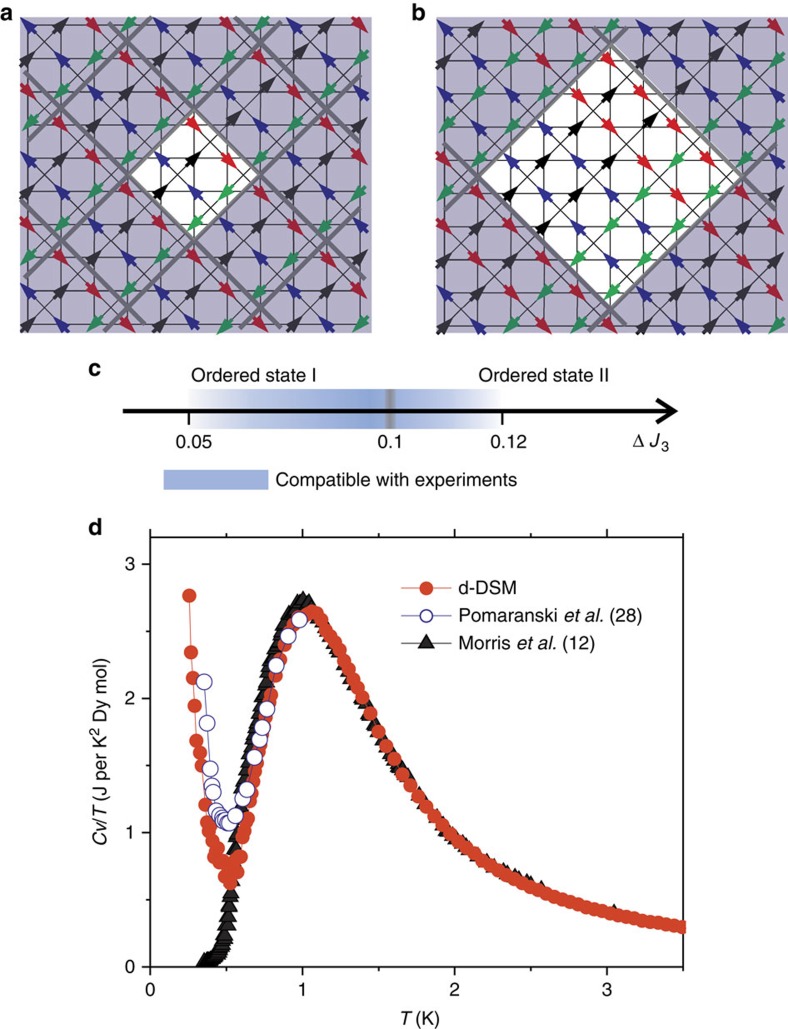
Ordered states of the model with distortions (d-DSM). Within the range of parameters fixed by the experimental susceptibility data, and depending on the difference Δ*J*_3_ between the third-nearest-neighbours exchange constant, the system orders into two possible states. (**a,b**) Cut along [100] of the two ordered states. The non-shaded regions correspond to the unit cells. (**c**) Ranges in Δ*J*_3_ where each state is observed. (**d**) The signature of this transition in the specific heat divided by temperature, compared with the results obtained for DTO in refs 12, 28.

Figure 5a,b shows cuts along [100] of the two ordered states obtained at low temperatures for different values of the exchange constants. For small values of *J*_2_, the relevant parameter that switches between the two types of order is the difference, Δ*J*_3_=*J*_3_*′−J*_3_. The scale in the middle (Fig. 5c) indicates, within the region compatible with the experiments (shaded in blue), the values of Δ*J*_3_ (for fixed average *J*_3_, and *J*_2_=0) where each type of order is seen. As expected, for low values of Δ*J*_3_, the ordered state is identical to the one originally found in the s-DSM. A simple description of this state (state I) is to consider it an antiferromagnetic arrangement of ferromagnetic chains of spins (indicated with the same colour in the figure) at axes rotated *π*/4 with respect to the original lattice. The second state (state II) can be thought as a simple extension of state I where the unit blocks are now pairs of ferromagnetic spin chains (also indicated with the same colour in the right hand side figure) ordered antiferromagnetically. In both cases, the non-shaded region in the figure corresponds to the unit cell.

These are the two states of lower energy that have been identified depending on the degree of asymmetry in the two *J*_3_. It is straightforward to conjecture that there is a wealth of possible intermediate states with energies differing by a few mK (in 1/*k*), constructed from all the possible periodic intercalations of different numbers of alternating single and double chains in each direction, or even of the alternation of bundles of a higher number of chains. This by itself has already the consequence that it will be extremely difficult for any experiment to probe equilibrium properties, but the situation in a real sample is even more complicated: the most likely experimental state is that the sample will have a distribution of tensions and distortions within its volume. This will lead to local changes in Δ*J*_3_, coming from the distortion-induced contribution to the two *J*_3_, which in turn will result into local energetic rearrangements of the possible states. No long-range ordered state will be favourable in the whole sample. The effect of tensions and distortions could also be intimately related with the experimental observation of freezing or glassy behaviour below temperatures of about 0.6 K^35^,^36^,^37^,^38^.

### Specific heat

Figure 5d shows the zero-field specific heat divided by temperature, calculated for the d-DSM with the same values used for Fig. 3, compared with the experimental data for DTO from refs 12, 28 (a finite size analysis of the simulated specific heat is shown in Supplementary Note 3 and Supplementary Fig. 2). Both experimental sets of data coincide at high temperatures, down to about 0.6 K below which the characteristic time of the system becomes extremely long and the results from ref. 28, taken over longer times, are believed to be closer to equilibrium. The upturn seen in this data has been interpreted as a possible sign of onset of long-range order. The specific heat calculated from the d-DSM matches the high temperature behaviour of the experiments and shows a sharp peak at a temperature close to 0.2 K, which corresponds to the ordering transition into state I or II (depending on the value of Δ*J*_3_). It would be tempting to associate this peak with the upturn of ref. 28, and match the two sets of data by making small adjustments to the exchange constants of the d-DSM. However, as discussed before, it might not be realistic to expect a homogeneous long-range ordered state in a real sample, but rather a wide range of local configurations and a multitude of energetically close excitations. In this case, features matching those seen in the specific heat data of ref. 28 have their origin in a Schottky-type peak consequence of this proliferation of states of very similar energies in this range of temperatures. A more definitive answer to this question would come from neutron scattering experiments performed under the same relaxation conditions as the specific-heat experiment.

## Discussion

In summary, in this work we present experiments performed in the two paradigmatic examples of spin-ice materials, DTO and HTO, where we have measured the angular dependence of susceptibility using precise alignment with the crystallographic axes, concentrating in particular in the polarization transition with field in the neighbourhood of [111]. We show that a generic feature of these two materials, absent from any theoretical spin-ice model in the literature, is a marked anisotropy in *χ*, which shows a doubled transition when the field is rotated towards [112]. This doubled transition had been previously observed in specific heat and magnetization experiments in DTO^23^,^26^, but not in HTO. We considered the possibility of distortions, a missing ingredient from the usual spin-ice models, and by performing the simplest possible analysis on their effect on the s-DSM we found that they provide a justification for the addition of interactions between second and two kinds of third nearest neighbours. This is a generic feature of distorted spin-ice materials, and applies even if the exchange terms for the specific material were negligible. There is a crucial difference in the current treatment of thermal vibrations to what already exists in the literature, which is a consequence of the presence of long-range interactions in this class of materials. Thermal vibrations usually just renormalize constants, but in the present case dipolar long-range interactions lead to the appearance of couplings that would not exist in the absence of vibrations. Thus, our treatment of the simple d-DSM leads to a more complex Hamiltonian, that resembles that of for example, ref. 19, but is not obtained in a purely empirical manner.

Provided with these ingredients we constructed a new model (the d-DSM) and explored its characteristics using Monte Carlo simulations. The new model reproduces the anisotropy found in the experiments with fields in the neighbourhood of [111], and, despite the fact that it was not built based on neutron scattering data or *Cv*, the *S*(*q*) calculated for the d-DSM reproduces the experimental results (see Supplementary Note 4 and Supplementary Fig. 3) and we have seen that the same is true for *Cv* (Fig. 5). More importantly, it predicts an intermediate magnetization state, thus explaining the hitherto unresolved issue of a doubled polarization transition with field close to the [111] direction, and also predicts the existence of different types of possible order at low temperatures when no field is applied. These results from the d-DSM are valid regardless the ultimate origin of the different interaction terms. The possibility that these further order terms could be a consequence of distortion would mean that the small tensions present in real samples could result in the absence of long-range order at low temperatures. This is probably the most surprising conclusion of this work, that distortions, which usually result in a relief of frustration, might be the reason why the system would remain disordered at very low temperatures.

Very recently, Henelius *et al*.^39^ have presented an empirical spin-ice Hamiltonian constructed to describe three main sets of experiments in DTO (the structure factor *S*(*q*), the specific heat *Cv* and the magnetization for *H* near [112]). The model Hamiltonians presented in both studies have several points of contact. In particular, both models predict the same possible ordered ground states for *H=*0 (those described in Fig. 5) and, crucially, the likelihood of a disordered ground state as a consequence of internal stress (in our case given by external strain, in theirs by impurities) and quasi-degeneracy. The similarity of these findings is remarkable taking into account that the core experiments used in their work and in ours are very different.

## Methods

### Experiments

For our experiments, single crystalline samples of DTO and HTO where grown using an optical floating zone in St. Andrews and Oxford, and they were oriented and cut into prisms of approximate dimensions (0.7 × 0.7 × 3) mm^3^, with the long axis oriented along [111]. The a.c. susceptibility was measured in a dilution refrigerator using two sets of detection coils, positioned so as to avoid cross-talk effects, and a drive coil. Each detection coil set comprises of two coils, connected antiparallel to each other. The drive coil is concentrically wound around the pick-up coils. The samples were placed in each of the upper pick-up coils and thermally grounded to the mixing chamber through silver and copper wires. A low-frequency and very low-amplitude excitation field of about 3 × 10^−5^ T r.m.s. was generated by the ac current in the drive coil, and the response detected by a lock-in amplifier. For accurate control of the external magnetic field, we used a bespoke triple-axis vector magnet capable of producing 9/1/1 T along *z*, *x* and *y*. Careful centring using the vector field capability allowed *θ* to be determined to an accuracy of 0.1° relative to the crystallographic axis [111].

### Theoretical analysis of the effect of distortions

A simpler effective Hamiltonian can be obtained from 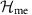 (equation (2)) by integrating out the phononic d.f.^26^. In the spin-ice model **S**_*i*_=*σ*_*i*_ **e**_*i*_, with *σ*=±1, which leads to important simplifications: since *σ*_*i*_^*2*^*=*1, the usual quartic terms become quadratic. Still, due to the long-range nature of dipolar couplings one encounters terms **F**_*ij*_ of any range, and the analysis of the effective magnetic model is rather complex. To compare the predictions of our model with those in the literature we have considered an expansion of the corrections to the full effective magnetic Hamiltonian up to third neighbours.

The effective magnetic model obtained reads


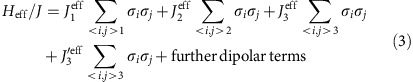


where <*i,j*>_*r*_ indicates *r*-th range neighbours. The couplings up to third neighbours are


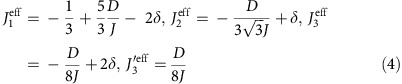


where *δ* is the contribution arising from the phonons, 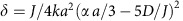. Further range terms are the unmodified dipolar interactions.

The first observation is that the crystal environment distinguishes two different third neighbour couplings in *H*_eff_: *J*_3_^eff^ and *J*_3_*′*^eff^ distinguish between third nearest neighbours connected through the lattice by a minimum of one or two other lattice sites respectively. One of them is sensitive to distortions, while the other is not. A second observation is that the relative strengths of all couplings in *H*_eff_ depend only on two microscopic parameters: *D/J* and *α/J*. Corrections of longer-range magnetic couplings can be done systematically, depending on the same microscopic parameters. We have checked that fourth-order corrections do not change the main results of this work.

It is not realistic to expect that a simple classical model such as the one presented here will give an accurate description of the real system at low temperatures. The presence of soft modes (speculated for spin-ice materials in ref. 40 or seen in Tb_2_Ti_2_O_7_ (ref. 41) could be underlying the relative success of such a treatment, and is worthy of being investigated.

### Monte Carlo simulations

For the d-DSM model we used the following Hamiltonian:


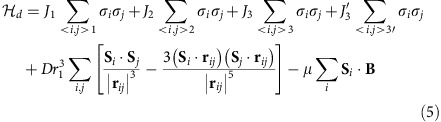


where the <*i,j*>_*3*_ and <*i,j*>_*3'*_ distinguish between third nearest neighbours connected through the lattice by a minimum of one or two other lattice sites respectively (see Fig. 1a).

We performed Monte Carlo simulations, using Ewald summations to take into account long-range dipolar interactions^42^,^43^ in a conventional cubic cell for the pyrochlore lattice. We simulated systems with *L × L × L* cells with periodic boundary conditions. Basic parameters were taken from ref. 19, including cell size, lattice parameter and magnitude of magnetic moments. Thermodynamic data as a function of field were collected using a variation of single spin-flip Metropolis algorithm. For Fig. 3 we chose a temperature of 0.4 K to avoid problems associated with hysteresis. For the specific heat data below 0.6 K we mixed the usual Metropolis with a dynamics that conserves defects for a certain number of Monte Carlo steps before letting the system relax (the algorithm is a variation of the one used in ref. 43 and is described in Supplementary Note 1). In the case of magnetization measurements at 0.1 K (Fig. 4), data at each field were collected after a slow annealing, averaging over five independent runs.

The average value of the third neighbour interactions was kept within the range specified in ref. 19 to retain compatibility with previous experiments in DTO. As an example, with the values of *J* used in Figs 3, 4, 5, we studied the transition to ferromagnetic ordering with field nearly along the [112] direction^19^,^21^. We obtained a transition temperature of ≈0.35 K, which could be justified considering angular deviations of <0.5° off perfect alignment.

Regarding the double peak near [111], we contrasted our simulations with the static magnetization data obtained in ref. 26, which shows narrower features than the a.c. susceptibility. The range fixed in Fig. 5 was determined looking for special key details: (i) a magnetization increase of only ∼0.025*μ* when the field is tilted from 0° to −5° (towards [112]) at a fixed field of 0.9 T, (ii) an increment for the first critical field of <0.06 T when tilting the field from 0° to −5° and (iii) a difference between the two critical fields at −5°of ≈0.1 T.

### Data availability

All relevant data are available from the authors.

## Additional information

**How to cite this article:** Borzi, R.A. *et al*. Intermediate magnetization state and competing orders in Dy_2_Ti_2_O_7_ and Ho_2_Ti_2_O_7_. *Nat. Commun.* 7:12592 doi: 10.1038/ncomms12592 (2016).

## Supplementary Material

Supplementary Information Supplementary Figures 1-3, Supplementary Notes 1-4 and Supplementary References

## References

[b1] Harris M. J., Bramwell S. T., McMorrow D. F., Zeiske T. & Godfrey K. W. Geometrical frustration in the ferromagnetic pyrochlore Ho2Ti2O7. Phys. Rev. Lett. 79, 2554–2557 (1997).

[b2] Bramwell S. T. & Gingras M. J. P. Spin ice state in frustrated magnetic pyrochlore materials. Science 294, 1495–1501 (2001). 11711667 10.1126/science.1064761

[b3] Siddharthan R. . Ising pyrochlore magnets: low-temperature properties, ice rules, and beyond. Phys. Rev. Lett. 83, 1854–1857 (1999).

[b4] den Hertog B. C. & Gingras M. J. P. Dipolar interactions and origin of spin ice in Ising pyrochlore magnets. Phys. Rev. Lett. 84, 3430–3433 (2000). 11019107 10.1103/PhysRevLett.84.3430

[b5] Bramwell S. T. . Spin correlations in Ho_2_Ti_2_O_7_: a dipolar spin ice system. Phys. Rev. Lett. 87, 047205 (2001). 11461643 10.1103/PhysRevLett.87.047205

[b6] Han Y. . Geometric frustration in buckled colloidal monolayers. Nature 456, 898–903 (2008). 19092926 10.1038/nature07595

[b7] Tanaka Y. . Lattice deformations induced by an applied magnetic field in the frustrated antiferromagnet HgCr_2_O_4_. J. Phys. Soc. Jpn 76, 043708 (2007).

[b8] Shokef Y., Souslov A. & Lubensky T. C. Order by disorder in the antiferromagnetic Ising model on an elastic triangular lattice. Proc. Natl Acad. Sci. 108, 11804–11809 (2011). 21730164 10.1073/pnas.1014915108 PMC3141946

[b9] Gingras M. J. Introduction to Frustrated Magnetism pp. 293–329Springer (2011).

[b10] Ryzhkin I. Magnetic relaxation in rare-earth oxide pyrochlores. J. Exp. Theor. Phys. 101, 481–486 (2005).

[b11] Castelnovo C., Moessner R. & Sondhi S. L. Magnetic monopoles in spin ice. Nature 451, 42–45 (2008). 18172493 10.1038/nature06433

[b12] Morris D. J. . Dirac strings and magnetic monopoles in the spin ice Dy_2_Ti_2_O_7_. Science 326, 411–414 (2009). 19729617 10.1126/science.1178868

[b13] Moessner R. & Sondhi S. L. Theory of the [111] magnetization plateau in spin ice. Phys. Rev. B 68, 064411 (2003).

[b14] Isakov S. V. . Magnetization curve of spin ice in a [111] magnetic field. Phys. Rev. B 70, 104418 (2004).

[b15] Jaubert L. D. C. . Three-dimensional Kasteleyn transition: spin ice in a [100] field. Phys. Rev. Lett. 100, 067207 (2008). 18352511 10.1103/PhysRevLett.100.067207

[b16] Melko R. G., den Hertog B. C. & Gingras M. J. Long-range order at low temperatures in dipolar spin ice. Phys. Rev. Lett. 87, 067203 (2001). 11497852 10.1103/PhysRevLett.87.067203

[b17] Fennell T. . Neutron scattering investigation of the spin ice state in Dy_2_Ti_2_O_7_. Phys. Rev. B 70, 134408 (2004).

[b18] Ruff J. P. C., Melko R. G. & Gingras M. J. P. Finite-temperature transitions in dipolar spin ice in a large magnetic field. Phys. Rev. Lett. 95, 097202 (2005). 16197244 10.1103/PhysRevLett.95.097202

[b19] Yavors'kii Taras . Dy_2_Ti_2_O_7_ spin ice: a test case for emergent clusters in a frustrated magnet. Phys. Rev. Lett. 101, 037204 (2008). 18764286 10.1103/PhysRevLett.101.037204

[b20] Ramirez A. P., Hayashi A., Cava R. J., Siddharthan R. & Shastry B. S. Zero-point entropy in ‘spin ice'. Nature 399, 333–335 (1999).

[b21] Higashinaka R., Fukazawa H., Deguchi K. & Maeno Y. Low temperature specific heat of Dy_2_Ti_2_O_7_ in the kagome ice state. J. Phys. Soc. Jpn 73, 2845–2850 (2004).

[b22] Hiroi Z., Matsuhira K., Takagi S., Tayama T. & Sakakibara T. Specific heat of kagome ice in the pyrochlore oxide Dy_2_Ti_2_O_7_. J. Phys. Soc. Jpn 72, 411–418 (2003).

[b23] Higashinaka R. & Maeno Y. Field-induced transition on a triangular plane in the spin-ice compound Dy_2_Ti_2_O_7_. Phys. Rev. Lett. 95, 237208 (2005). 16384340 10.1103/PhysRevLett.95.237208

[b24] Sato H. . Ferromagnetic ordering on the triangular lattice in the pyrochlore spin-ice compound Dy_2_Ti_2_O_7_. J. Phys. Condens. Matter 18, L297–L303 (2006).

[b25] Sakakibara T. . Observation of a liquid–gas-type transition in the pyrochlore spin ice compound Dy_2_Ti_2_O_7_ in a magnetic field. Phys. Rev. Lett. 90, 207205 (2003). 12785926 10.1103/PhysRevLett.90.207205

[b26] Sato H. . Field-angle dependence of the ice-rule breaking spin-flip transition in Dy_2_Ti_2_O_7_. J. Phys. Condens. Matter 19, 145272 (2007).

[b27] Grigera S. A. . An intermediate state between the kagome-ice and the fully polarized state in Dy_2_Ti_2_O_7_. Pap. Phys. 7, 070009 (2015).

[b28] Pomaranski D. . Absence of Pauling's residual entropy in thermally equilibrated Dy_2_Ti_2_O_7_. Nat. Phys. 9, 353–356 (2013).

[b29] Gómez Albarracín F. A., Cabra D. C., Rosales H. D. & Rossini G. L. Spin-phonon induced magnetic order in the kagome ice. Phys. Rev. B 88, 184421 (2013).

[b30] Penc K., Shannon N. & Shiba H. Half-magnetization plateau stabilized by structural distortion in the antiferromagnetic Heisenberg model on a pyrochlore lattice. Phys. Rev. Lett. 93, 197203 (2004). 15600874 10.1103/PhysRevLett.93.197203

[b31] Vekua T., Cabra D. C., Dobry A., Gazza C. & Poilblanc D. Magnetization plateaus induced by a coupling to the lattice. Phys. Rev. Lett. 96, 117205 (2006). 16605861 10.1103/PhysRevLett.96.117205

[b32] Snyder J. . Low-temperature spin freezing in the Dy_2_Ti_2_O_7_ spin ice. Phy. Rev. B 69, 064414 (2004).

[b33] Jaubert L. D. & Holdsworth P. C. Signature of magnetic monopole and Dirac string dynamics in spin ice. Nat. Phys. 5, 258–261 (2009).

[b34] Slobinsky D. . Unconventional magnetization processes and thermal runaway in spin-ice Dy_2_Ti_2_O_7_. Phys. Rev. Lett. 105, 267205 (2010). 21231712 10.1103/PhysRevLett.105.267205

[b35] Snyder J. . How ‘spin ice' freezes. Nature 413, 48–51 (2001). 11544520 10.1038/35092516

[b36] Orendáč M. . Magnetocaloric study of spin relaxation in dipolar spin ice Dy_2_Ti_2_O_7_. Phys. Rev. B 75, 104425 (2007).

[b37] Klemke B. . Thermal relaxation and heat transport in the spin ice material Dy_2_Ti_2_O_7_. J. Low Temp. Phys. 163, 345–369 (2011).

[b38] Yaraskavitch L. R. . Spin dynamics in the frozen state of the dipolar spin ice material Dy_2_ Ti_2_O_7_. Phys. Rev. B 85, 020410 (2012).

[b39] Henelius P. . Refrustration and competing orders in the prototypical Dy_2_Ti_2_O_7_ spin ice material. Phys. Rev. B 93, 024402 (2016).

[b40] Ramirez A. P. . Geometrical frustration, spin ice and negative thermal expansion—the physics of underconstraint. Physica B 280, 290–295 (2000).

[b41] Fennell T. . Magnetoelastic excitations in the pyrochlore spin liquid Tb_2_Ti_2_O_7_. Phys. Rev. Lett. 112, 017203 (2014). 24483925 10.1103/PhysRevLett.112.017203

[b42] Melko R. G. & Gingras M. J. Monte Carlo studies of the dipolar spin ice model. J. Phys. Condens. Matter 16, R1277–R1319 (2004).

[b43] Borzi R. A., Slobinsky D. & Grigera S. A. Charge ordering in a pure spin model: dipolar spin ice. Phys. Rev. Lett. 111, 147204 (2013). 24138269 10.1103/PhysRevLett.111.147204

