# Supplementary material for: Intermediate magnetization state and competing orders in Dy2Ti2O7 and Ho2Ti2O7
Source: Nat Commun. 2016 Aug 25;7:12592. doi: 10.1038/ncomms12592 (PMC5007346; doi:10.1038/ncomms12592)
Supplement: Supplementary Information — Supplementary Figures 1-3, Supplementary Notes 1-4 and Supplementary References [file ncomms12592-s1.pdf]

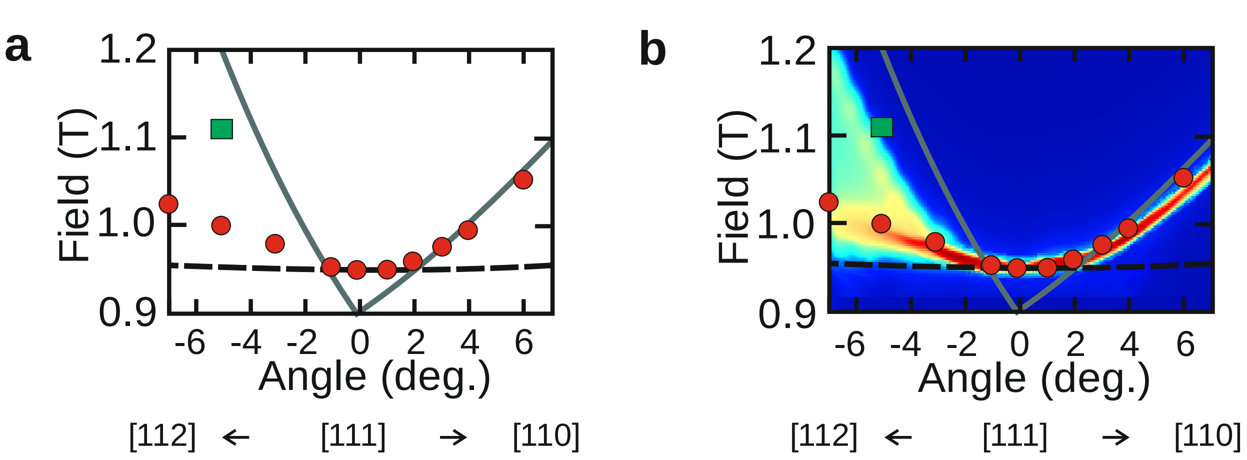

Supplementary Figure 1 | Comparison between the experimental field-angle phase diagram and the g-DSM model. **(a)** The phase diagram from Sato et al. [1] determined using magnetisation experiments (data from inset of Fig. 4 of [1]). The symbols represent the critical field measured with circles (red) for Hc_1 and the square (green) for Hc_2. The solid line corresponds to a hypothetical transition line, as calculated from the nearest neighbours model. **(b)** The diagram in **(a)** has been overlaid on top of a contour plot of the susceptibility calculated from the d-DSM (data from Fig. 3 of the main text). We can see that there is good coincidence between the experiments and the model.

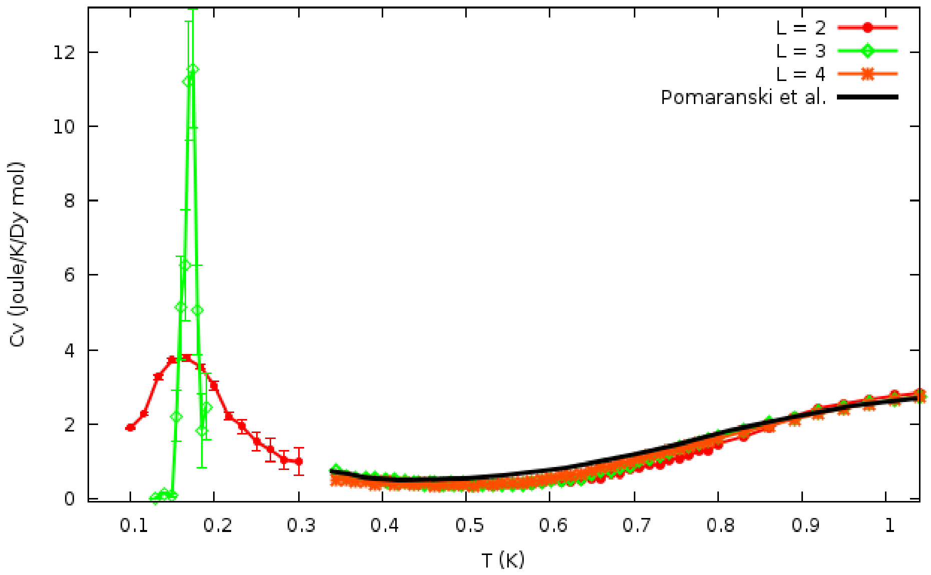

Supplementary Figure 2 | Finite size scaling of the specific heat in the g-DSM model. Specific heat calculated for the g-DSM model for two temperature ranges and different system sizes as indicated in the figure. The black line corresponds to the experimental data by Pomaranski et al. [2]

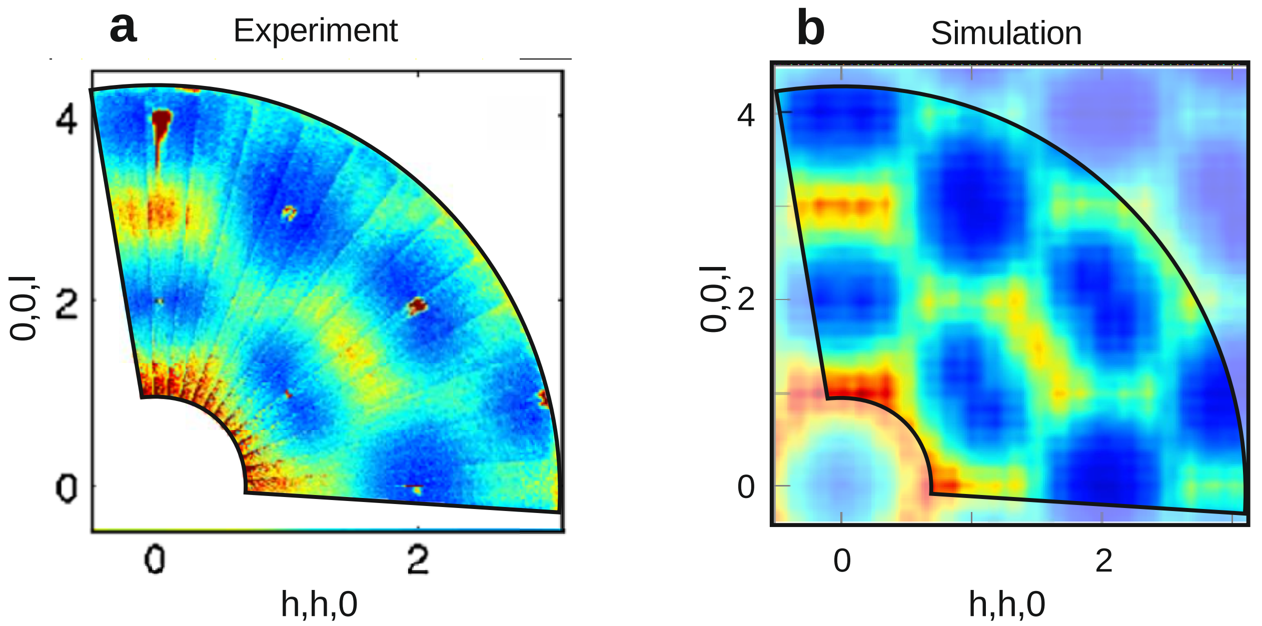

Supplementary Figure 3 | Comparison between the experimental and simulated structure factors. (**a**) the experimental *S(q)* of $Dy_2Ti_2O_7$ as a function of the wavevector (from [3]) and (**b**) the *S(q)* calculated from the d-DSM, both at 300mK.

Supplementary Note 1: Monte Carlo simulations

In order to simulate the specific heat at low temperatures we have implemented a single spin flip dynamics, but different from the conventional in that we use of the Conserved Monopoles Algorithm (CMA) to reach equilibration at low temperatures in reasonable simulation times. An advantage of the CMA is that it can also be used in the presence of a magnetic field. A description of this algorithm can be found in [4] where it was introduced, and subsequently it has also been discussed and applied by other authors (see e.g. [5]). The CMA works in a statistical ensemble of conserved number of defects to the ice rule, which are free to propagate in the system. It can be shown that even a negligible density of defects (two in a lattice of thousands of sites) is enough, by lowering the barriers between states, to speed up the simulations substantially. For example (as shown in [4]) the CMA applied to the DSIM at zero field reproduces the results of Melko et al. [6].

We introduced in this work a small improvement to the CMA algorithm. In order to avoid any interference of the artificial defects in the values of the thermodynamic variables, before collecting one sample we remove the constraint and allow the defects to disappear while we let the system evolve for some steps. After the data is collected, we reintroduce a pair of defects and let the system continue its evolution. The model is hybrid, in that it only introduces the CMA after checking that the density of monopoles at a given temperature falls below a

threshold. We have checked the code by reproducing again the phase transition towards the order state observed at 180 mK in the dipolar model by Melko and collaborators [6].

Supplementary Note 2: Selection of the exchange constants for the model.

It is quite remarkable that a simple model for distortion gives an expression that, for a given δ and J_1, provides values for J_2 and for both J_3's that lead to a Hamiltonian compatible with most of the experiments discussed in [3] where the J values were chosen in an ad-hoc fashion. This can be seen noting that the values of J_2 and $J_3^{av} = (J_3^{\ 1} + J_3^{\ 2})/2$ predicted by the theoretical analysis discussed in the main text can be made to fit within the first range delimited in [3]: -0.20 K < J_2' < 0 K; 0.019 K < J_3' < 0.026 K. (Note that due to a geometrical factor implicitly taken into account in our model, our nominal value of J_2 has the opposite sign and with a modulus three times bigger than that in ref. 17, i.e. $J_2' = -1/3\ J_2$). By choosing a bigger δ, we could even find a modest peak splitting for negative θ and no splitting in the opposite direction, for a limited value of θ.

As a further refinement, and recognising that ours is just a minimal model, we used the parameters quoted in the text, which preserve the main constraints: nonnegative values for J_2 and J_3^{av}, with different $J_3^{\ 1}$ and $J_3^{\ 2}$, but which depart from the calculated relations. These parameters increased the peak splitting, extended the range of theta in which the double peak was observed (up to $\theta \sim 8$ deg in the simulations), and improved the quantitative comparison of our magnetisation curves with those in [1] (the low value of the magnetisation at fields just below the jumps was a key piece of information). We included, for comparison, a figure (supplementary Figure 1) that shows the experimental phase diagram determined measuring magnetisation by Sato el al. [1] and the same diagram overlaid on top of the susceptibility contour plot of the d-DSM (data from our Fig. 3 in the main text). Note that our sign convention for the angle is opposite to that of Sato et al. [1].

One (apparent) drawback we observed was that while we improved the similarity of the simulated double peak feature with the experimental data, the new set of J's led to an unexpected increase of Cv at low T. Afterwards we realised that the new feature near ~0.4K in Cv was in agreement with that experimentally observed in [2] (see e.g. Supplementary Figure 2). It may be worth repeating that no effort was made to tune the J values in order to make our specific heat look like that in [2]. Furthermore, as can be seen in the Supplementary Note 4 and Supplementary Figure 3, this set of exchange constants also gives a very good account of the experimental $S(q)$.

Supplementary Note 3: Finite size scaling of the specific heat at zero field.

We provide a finite size effect (FSE) study at H=0. We constrained it to small sizes, due to the computer time demanded by this study (see Supplementary Figure 2)

We concentrate on two important temperature ranges:

a) Temperatures above ~ 0.4 K: This range is the main focus of our work. Recent experimental data for the specific heat is available down to a temperature of approximately 0.36K [2] (the lowest temperature of the data in Fig. 5 of the main text). Since we compare it with our equilibrated data, it is an implicit assumption of our work that this experimental specific heat is properly equilibrated.

   It is reasonable to wonder if the rise we observe in Cv/T in our simulations (bottom of Fig. 5 of the main text) is due to the imminent phase transition and thus affected by finite size effects. If so, the coincidence between our simulations and the experimental Cv/T would be merely accidental. The figure above, including data for L = 2, 3, 4 and the experimental data from [2] shows that in the regime where Cv turns up (between ~ 0.5 and 0.36 K) the variations due to finite size effects are not very significant and it is sensible to compare our simulated data with the experimental results. (A small finite size effect between 0.5 and 0.8 K may indicate that the coincidence can be even better in the thermodynamic limit).

b) Though we have been interested in the possible ground states predicted by our model, the phase transition occurring at low temperature has yet no experimental counterpart, and, as we mentioned, its nature is not one of the main concerns of this work. The figure shows some preliminary low temperature data, which are compatible with a first order transition. Indeed, the maximum of Cv near 0.17 K increases, within error, like the volume of the system when going from L=2 to that L=3.

Measuring the specific heat with Ewald summations to take into account dipolar interactions, and using the conserved monopoles algorithm at low temperatures for L =3 with the statistics observed in the figure below (green curve) demanded 35 copies of a program running for a month (35x30x24 h = 25200 hours of computer time). To be more specific, it demanded 11 temperature points with $3\text{x}10^7$ MCS per point, per independent run. Given time and computer time limitations, we have not tried to measure the finite size effect for L =4 or bigger lattices at very low temperature.

Supplementary Note 4: Calculation of the structure factor S(q)

We calculated the structure factor predicted by the d-DSM model introduced in this work. We use

$$S(q) = \frac{[f(|\mathbf{q}|)]^2}{N} \sum_{i,j} \langle \sigma_i \sigma_j \rangle (\mathbf{e}_i^{\perp} \cdot \mathbf{e}_j^{\perp}) e^{\mathbf{q} \cdot r_{ij}}$$

where the $<\sigma_i \sigma_j>$ are the correlations between Ising spins at sites i and j, $\mathbf{e}_i^{\perp}$ is the component of the quantisation direction at site i perpendicular to the scattering vector $\mathbf{q}$, N is the number of spins and $f(|\mathbf{q}|)$ is the magnetic form factor for Dy^{3+} extracted from [7].

We find that the $S(q)$ calculated from our model is compatible with the existing experimental results. It is important to remark that no effort was put before hand to enforce this compatibility: the J values were optimised in relation to the double feature observed in the susceptibility measurements that we describe in the text.

The Supplementary Figure 3 shows a comparison between the experimental data from [3] at T = 300 mK and our calculations for the same temperature in a lattice of $L = 6$ and over 200 averaged copies, each obtained after a zero field cooling:

Some noteworthy points of comparison are: i) The presence in both the experimental data and the simulation of hexagonal loops of diffuse scattering running along the Brillouin zone boundaries. ii) The presence of pinch points characteristic to spin-ice and iii) The presence of regions of intense scattering around (0,0,3) and (3/2, 3/2, 3/2). The narrow Bragg peaks, due to the lattice structure, are absent in our simulations.

Supplementary References

1. Sato, H., Matsuhira, K., Sakakibara, T., Tayama, T., Hiroi, Z., & Takagi, S. Field-angle dependence of the ice-rule breaking spin-flip transition in $Dy_2Ti_2O_7$. *J. Phys. Cond. Matt.* **19**, 145272 (2007).
2. Pomaranski, D. *et al.* Absence of Pauling's residual entropy in thermally equilibrated $Dy_2Ti_2O_7$. *Nature Physics* **9**, 353-356 (2013).
3. Yavors'kii, Taras, *et al*. $Dy_2Ti_2O_7$spin ice: a test case for emergent clusters in a frustrated magnet. *Phys. Rev. Lett.* **101**, 037204 (2008).
4. Borzi, R. A., D. Slobinsky, & S. A. Grigera. Charge Ordering in a Pure Spin Model: Dipolar Spin Ice. *Phys. Rev. Lett.* **111,** 147204 (2013).
5. Xie, Y. L., Du, Z. Z., Yan, Z. B., & Liu, J. M. Magnetic-charge ordering and phase transitions in monopole-conserved square spin ice. *Scientific reports*, **5**. 15875 (2015).
6. Melko, R. G., den Hertog, B. C., & Gingras, M. J. Long-range order at low temperatures in dipolar spin ice. *Phys. Rev. Lett.*, **87**, 067203 (2001).
7. Brown, P. J. in International Tables for Crystallography vol C (ed AJC Wilson.) Ch. 4.4.5, p 391 (Kluwer Academic, Dordrecth, Holland,1995).
